# Pharmacokinetics, radiation dosimetry, acute toxicity and automated synthesis of [^18^F]AmBF_3_-TATE

**DOI:** 10.1186/s13550-020-0611-9

**Published:** 2020-03-19

**Authors:** Joseph Lau, Jinhe Pan, Etienne Rousseau, Carlos F. Uribe, Sudhakara Reddy Seelam, Brent W. Sutherland, David M. Perrin, Kuo-Shyan Lin, François Bénard

**Affiliations:** 1 Department of Molecular Oncology, BC Cancer, Vancouver, BC Canada; 2 Department of Functional Imaging, BC Cancer, Vancouver, BC Canada; 3 Department of Experimental Therapeutics, BC Cancer, Vancouver, BC Canada; 4 Department of Chemistry, University of British Columbia, Vancouver, BC Canada

**Keywords:** Positron emission tomography, Neuroendocrine tumors, AmBF_3_-TATE, Fluorine-18, Dosimetry, Toxicology

## Abstract

**Introduction:**

[^18^F]AmBF_3_-TATE is a somatostatin agonist that selectively binds to somatostatin receptor subtype 2 (SSTR2). For clinical translation, pharmacokinetics, radiation dosimetry, and acute toxicity of [^18^F]AmBF_3_-TATE were assessed with good laboratory practice (GLP) standards.

**Methods:**

ICR mice were intravenously administered 0.8–2.0 MBq of [^18^F]AmBF_3_-TATE, with one group pre-injected with 100 μg of [^19^F]AmBF_3_-TATE 30 min before radiopharmaceutical administration to assess uptake specificity. The mice were euthanized at 0.5, 1, 2, or 4 h post-injection (p.i.). Blood and tissues were collected, weighed, and counted on a gamma counter to determine percentage injected dose per gram (%ID/g). Dosimetry was calculated based on biodistribution data using the mouse and human phantoms included in OLINDA. Acute toxicity was assessed in Sprague-Dawley rats at the dose of 0.742 mg/kg [^19^F]AmBF_3_-TATE, with a 14-day observation/recovery period. Blood chemistry parameters, gross, and histopathology were evaluated. Body weight change and food consumption were monitored. The production of [^18^F]AmBF_3_-TATE was automated on a Trasis AllinOne synthesis module.

**Results:**

[^18^F]AmBF_3_-TATE was cleared through the renal and hepatobiliary pathway. At 1 h p.i., the pancreas (F, 15.7 ± 3.72 and M 14.3 ± 1.61 %ID/g), stomach (F, 15.3 ± 3.63 and M, 19.0 ± 3.49 %ID/g), and lungs (F, 9.26 ± 2.24 and M, 6.17 ± 6.04 %ID/g) were the organs with the highest specific uptake. Pre-injection with [^19^F]AmBF_3_-TATE significantly reduced pancreatic uptake (F, 0.13 ± 0.03 and M, 0.18 ± 0.09 %ID/g) at 1 h p.i. For dosimetry extrapolated to the average adult human, the bladder (0.027–0.030 mGy/MBq), pancreas (0.018–0.028 mGy/MBq), and lungs (0.006–0.013 mGy/MBq) are expected to receive the highest doses. No test-item related effects were observed upon evaluation of clinical observations, body weights, food consumption, clinical pathology, gross pathology, and histopathology for acute toxicity. [^18^F]AmBF_3_-TATE was produced at activity yields of 15.6 ± 4.59 GBq, average molar activity of 435 ± 162 GBq/μmol, and radiochemical purity of 98.0 ± 1.73% with the automated synthesizer.

**Conclusion:**

[^18^F]AmBF_3_-TATE binds specifically to SSTR2. At 1000× clinical dose, [^19^F]AmBF_3_-TATE was well tolerated with no treatment-related adverse effects.

## Introduction

Neuroendocrine tumors (NET) are low-incidence cancers that originate from the neuroendocrine system [1]. NETs can arise from many anatomical sites with approximately 70% of cases derived from gastrointestinal origin [1]. Because NETs tend to be indolent and slow growing, patients can be asymptomatic for years for non-secreting tumors. Consequently, a significant number of patients present with high tumor burden or metastatic disease at diagnosis [1]. Radiolabeled somatostatin analogs have been used as nuclear imaging agents as NETs commonly overexpress somatostatin receptor subtype 2 (SSTR2). Clinically used SSTR2-targeting positron emission tomography (PET) agents include, but are not limited to, [^68^Ga]Ga-DOTA-TATE, [^68^Ga]Ga-DOTA-TOC, and [^68^Ga]Ga-NODAGA-JR11 [2, 3]. These somatostatin analogs can be radiolabeled with beta-emitting radionuclides (e.g., lutetium-177) for peptide receptor radionuclide therapy [4, 5].

Recently, the Society of Nuclear Medicine and Molecular Imaging (SNMMI) penned an open letter to the US Food and Drug Administration (FDA) highlighting the shortage of good manufacturing practice (GMP)-grade germanium-68/gallium-68 generators [6]. In the USA, the preparation of [^68^Ga]Ga-DOTA-TATE is approved with GalliaPharm® generators (Eckert & Ziegler), which expires after 550 elutions or 1 year and with Galli Eo® generators (IRE ELiT), which expires after 450 elutions or 1 year. Despite the availability of germanium-68, the production capacity of these generators remains limited. Consequently, many academic sites within the USA are unable to supply [^68^Ga]Ga-DOTA-TATE, extending wait times for patients [6]. The demand for [^68^Ga]Ga-DOTA-TATE is expected to increase given the recent FDA approval of [^177^Lu]Lu-DOTA-TATE. To alleviate the shortage, the SNMMI proposed temporary exemptions to elution limit, approval of generators from different manufacturers, and adoption of cyclotron produced gallium-68 [6]. Whether or not these solutions will ensure adequate coverage for clinical demands remains to be seen.

**Fig. 1:**
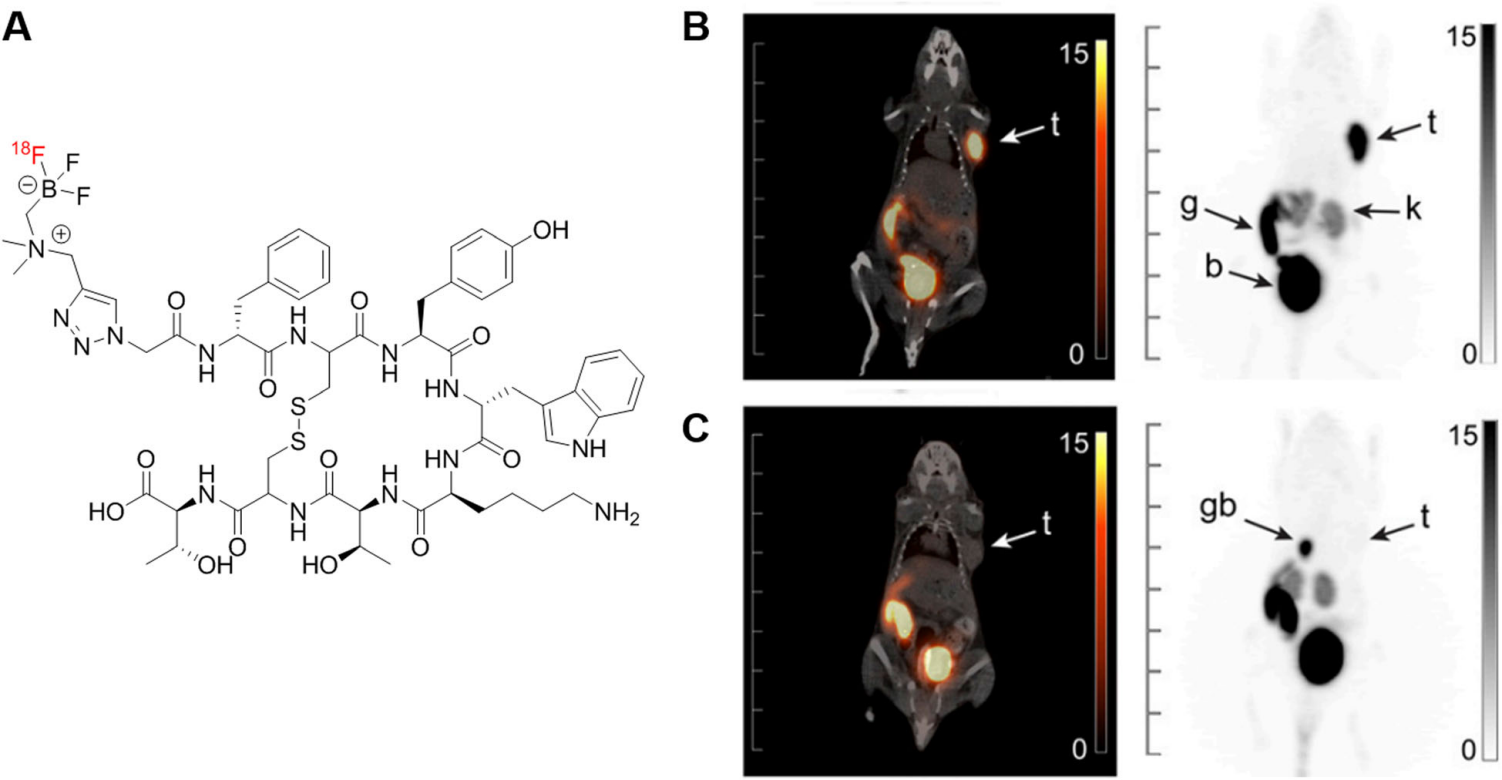
**a** Chemical structure of [^18^F]AmBF_3_-TATE. **b** Baseline PET/CT and PET maximum intensity projection image of [^18^F]AmBF_3_-TATE acquired at 60-min post-injection in mice bearing AR42J exocrine pancreatic tumor and **c** with 15 min pre-injection of Ga-DOTA-TATE for blockade. *t* tumor, *g* gut, *k* kidney, *b* bladder, *gb* gallbladder. Figure adapted with permissions from Liu et al. Copyright 2014 Society of Nuclear Medicine and Molecular Imaging

An advantageous long-term solution may be the adoption of trifluoroborate-based somatostatin analogs [7]. Previously, our laboratory developed [^18^F]AmBF_3_-TATE, a somatostatin agonist that can be radiolabeled with fluorine-18 using a one-step isotope exchange reaction (Fig. 1) [8]. Fluorine-18 has advantages over gallium-68 including availability in large quantities from cyclotron (allowing imaging of more patients per synthesis), optimal imaging properties with a shorter positron range, and longer half-life that permits distribution from a centralized radiopharmacy to other institutions and imaging at later time points for optimal contrast. AMBF_3_-TATE has better binding affinity to SSTR2 than Ga-DOTA-TATE (*K*_i_, 0.13 ± 0.03 vs. 0.7 ± 0.2 nM) [8]. Preclinical PET imaging data with [^18^F]AmBF_3_-TATE in AR42J tumor xenograft-bearing mice showed rapid clearance from blood and liver, predominant urinary excretion, and high tumor uptake and high tumor-to-background contrast ratios [8].

For anticipated clinical translation, we evaluated the pharmacokinetics, radiation dosimetry, and acute toxicity of [^18^F]AmBF_3_-TATE in rodent models in accordance with good laboratory practice (GLP) regulations. Moreover, we implemented the synthesis of [^18^F]AmBF_3_-TATE on an automated synthesis module.

## Materials and methods

### Biodistribution study

ICR mice (40 females and 40 males; 4–5 weeks old) were purchased from Envigo. Following an institutional quarantine, mice were given a 1-week acclimatization period before randomization into treatment or blocking groups. Health statuses of the mice were monitored daily. [^19^F]AmBF_3_-TATE was custom ordered as a GLP qualified peptide (≥ 95% purity) from AmbioPharm. [^18^F]AmBF_3_-TATE was synthesized at the cyclotron radiopharmacy at BC Cancer, Vancouver, according to published procedures [8]. The mice (five groups: 0.5, 1, 2, 4, and 1 h with blocking; eight females and eight males per group) were intravenously injected with 100–200 μL of [^18^F]AmBF_3_-TATE at a dose of 0.5–2.0 MBq through the lateral tail vein. Radioactivity measurements were performed on a Capintec CRC-25R dose calibrator. One group of mice received an intraperitoneal blocking dose of [^19^F]AmBF_3_-TATE (100 μg reconstituted in 200 μL United States Pharmacopoeia (USP)-grade saline from Baxter) 30 min before radiopharmaceutical administration. Following uptake periods of 0.5, 1, 2, or 4 h, mice were euthanized by CO_2_ asphyxiation. Blood was promptly drawn via cardiac puncture, and any urine released was collected. Organs of interest (Additional file 1: Tables S1 and S2) were harvested, rinsed with PBS, patted dry, and collected into pre-weighed scintillation tubes. The samples were counted on a Perkin Elmer 2480 WIZARD2 gamma counter to determine the amount of radioactivity in liquids/tissues. After counting, the tubes were re-weighed to determine the mass by subtraction. Raw data was processed and presented as percent injected dose per gram of tissue (%ID/g). All applicable institutional and/or national guidelines for the care and use of animals were followed, and the study protocol was approved by the institutional Research Ethics Board.

### Dosimetry

The biodistribution data of [^18^F]AmBF_3_-TATE was decayed from injection to its corresponding time point. Mono-exponential and bi-exponential fits, for each of the organs, were performed in Python. The best fit was chosen based on the coefficient of determination (*R*^2^) and the residuals of the fit (Additional file 1: Figs. S1 and S2). Residence times were calculated from integration of the fitted time-activity-curves, scaled to the organ mass of the model chosen for dosimetry calculation (NURBS for human and 25 g MOBY for mice). The results were used as input in OLINDA v.2.0 (Hermes Medical Solutions) to obtain mouse dosimetry and extrapolated human dosimetry [9–12].

### Acute toxicity

Acute toxicity studies were performed by Nucro-Technics, Ontario Canada. Sprague-Dawley rats were ordered from Charles River (30 males and 30 females; 7–8 weeks old). After an acclimatization period of 8 days, rats were randomized into one of two studies: interim and main. Rats were intravenously administered 0.742 mg/kg of [^19^F]AmBF_3_-TATE (reconstituted with USP-grade saline from B Braun) or vehicle control. Animals were monitored twice daily during the recovery period for clinical signs. Animals in the interim study (20 males and 20 females) were euthanized by isoflurane 1 day post-treatment, while those in the main study (10 males and 10 females) were euthanized by isoflurane 14 days post-treatment. Prior to necropsy, blood samples were evaluated for hematological and chemical parameters, and urine samples were subjected to urinalysis. In addition to experimental control, baseline data used for comparison for clinical pathology parameters were taken from Nucro-Technics’ historical database accounting for sex and age of the animals. Gross pathology analysis was performed immediately after euthanasia; organs of interest were harvested, fixed and preserved in 10% formalin, embedded in paraffin wax, and sectioned and stained by hematoxylin and eosin (H&E). Tissues were examined microscopically by a pathologist. Body weights were recorded prior to dose, prior to necropsy, and weekly during the recovery period. Weekly food consumption was monitored for animals in the main study. Studies adhered to all applicable institutional and/or national guidelines for the care and use of animals.

### Automation

#### Reagents

[^19^F]AmBF_3_-TATE was ordered from AmbioPharm Inc. Saline of USP grade and sterile water for injection were purchased from Baxter. Dimethylformamide (DMF), pyridazine, potassium hydrogen difluoride, and formic acid were purchased from Sigma-Aldrich. Hydrochloric acid for trace analysis was purchased from Honeywell. Ethanol was purchased from Commercial Alcohols. Acetonitrile was purchased from ThermoFisher Scientific. Ascorbic acid injection was purchased from Sandoz.

No-carrier-added ^18^F-fluorine was produced by bombardment of [^18^O]H_2_O (Huayi Isotopes Co.) with 18-MeV protons. Target irradiations were performed on a TR-19 cyclotron system (Advanced Cyclotron Systems Inc.) at the Department of Functional Imaging, BC Cancer Vancouver, Canada.

#### Setup of automated synthesizer

**Fig. 2:**
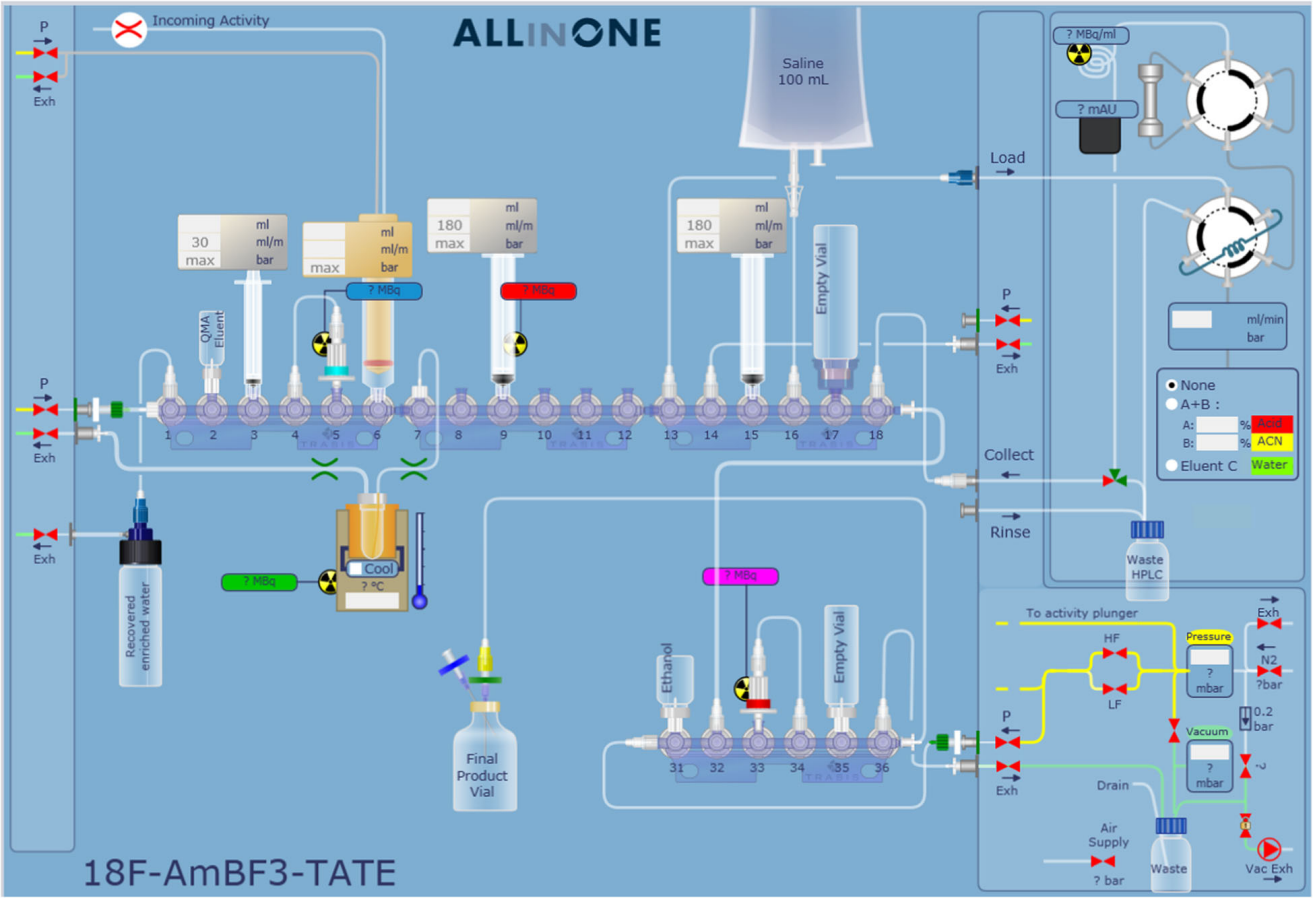
Setup of the synthesis cassette to produce [^18^F]AmBF_3_-TATE with integrated HPLC purification on Trasis AllInOne module

Production of [^18^F]AmBF_3_-TATE was conducted on a Trasis AllInOne module with 36 manifold actuators and an integrated HPLC system equipped with a Phenomenex Luna C-18 semi-preparative column (100 Å, 10 μm, 10 mm × 250 mm). The cassette setup is shown in Fig. 2 with tubing connections, syringes, and other disposables. The cassettes were pre-assembled in a cleanroom using disposable materials supplied by Trasis. Luer lock syringes (Becton Dickinson) were secured at Positions 3 (3 mL), 9, and 15 (10 mL). A Sep-Pak® Light Accell^TM^ Plus QMA-Cartridge (Waters) preconditioned with 10 mL saline followed by 20 mL DI water and a Sep-Pak® Light C-18 Cartridge (Waters) preconditioned with 10 mL ethanol followed by 20 mL DI water were installed on Positions 5 and 33, respectively.

#### Process description

The target water containing ^18^F-fluoride was transferred onto the module via a 20-mL syringe body without a plunger attached to Valve 6. The irradiated [^18^O]H_2_O was passed through the QMA-cartridge in Position 5 into a vessel to trap the ^18^F-fluoride onto the QMA-cartridge, while the enriched water was recovered in vacuo. The ^18^F-fluoride was eluted from the cartridge with 300 μL saline (Position 2) using the syringe in Position 3 into the Reaction Vessel (Position 7) preloaded with an aqueous solution of 1 M pyridazine and 2% HCl (pH 2, 45 μL), DMF (45 μL), 5 mM potassium bifluoride (4 μL), and [^19^F]AmBF_3_-TATE precursor (200 nmol). The reaction proceeded for 15 min at 85 °C under vacuum (− 1000 mbar). The reaction mixture was diluted with a solution of saline and 2.5 mg/mL ascorbic acid (5.8 mL) in Position 16 and loaded into the HPLC injection loop using the syringe in Position 9. [^18^F]AmBF_3_-TATE was purified by HPLC (Luna C-18 Column, 100 Å, 10 μm, 10 mm × 250 mm, 23% acetonitrile with 100 ppm ascorbic acid, flow rate of 4.5 mL/min). The product peak was collected into a 50 mL vial in Position 17 and diluted with the solution of saline and 2.5 mg/mL ascorbic acid (35 mL) in Position 16. The mixture was passed through a C-18 cartridge in Position 33 using the syringe in Position 15. The C-18 cartridge was rinsed with a solution of saline and 2.5 mg/mL ascorbic acid (10 mL) in Position 16, then [^18^F]AmBF_3_-TATE was eluted by ethanol (1 mL) in Position 31 into a 20 mL vial in Position 35 and diluted with a solution of saline and 2.5 mg/mL ascorbic acid (16 mL) in Position 16 using the syringe in Position 15. The solution was passed through a 0.2-μm sterilizing filter (Merck Millipore) into a pre-sterilized product vial.

#### Quality control (QC)

Filter integrity was assessed using a bubble point test. The pH value of the formulation was determined by pH indicator strips (EMD Millipore). Residual solvents in the injection solution were measured by gas chromatography using a 7820A GC System with GC Chem Station software (Agilent Technologies). The radionuclidic identification was determined by half-life measurement using a Capintec CRC-25R dose calibrator. The concentration of ascorbic acid was determined by colorimetric assay using ascorbic acid test strips (Macherey-Nagel). The radionuclidic purity was measured by gamma spectroscopy using a high-purity germanium (HPGe) radiation detector with multichannel analyzer (Canberra). The HPGe detector was operated with the Canberra Genie 2.0 software. The limulus amebocyte lysate (LAL) test was conducted on an Endosafe® PTS (Charles River Laboratories). Finally, a sample of the product formulation was tested for sterility post-release by an independent institution (Nucro-Technics) using direct inoculation in accordance with the USP monographs.

Calibrations of the HPLC systems were conducted by injection of [^19^F]AmBF_3_-TATE (0.01, 0.05, 0.1, 0.5, and 1 μg), DMF (0.04, 0.2, 0.4, 2, and 4 μg), and pyridazine (0.04, 0.2, 0.4, 2, and 4 μg) reference standards. HPLC analysis was performed on an Agilent HPLC system equipped with a model 1200 quaternary pump, a model 1200 UV absorbance detector (set at 220 nm), a Bioscan NaI scintillation detector, and an analytical column (Luna C18, 5 μm, 4.6 mm × 250 mm) purchased from Phenomenex. The operation of Agilent HPLC systems was controlled using the Agilent ChemStation software. For analysis, a multi-step gradient was applied using Solvent A (0.1% formic acid in water) and Solvent B (0.1% formic acid in acetonitrile): Solvent A in the first 5 min, then transitioning linearly to 76% A and 24% B over 1 min, then held at 76% A and 24% B for 5 min, then transitioning to 20% A and 80% B and over 3 min, then to solvent A over 1 min, and finally held at solvent A for 5 min (flow rate = 2 mL/min).

### Statistical analyses

For biodistribution data, uptake in %ID/g is presented as mean ± standard deviation. To examine target specificity, mice from 1-h time point and mice from 1-h time point pre-treated with the blocking agent were subjected to Student’s *t* tests (one-tail, unpaired) using Microsoft Excel 2007v. Group differences were considered significant at the level of *p* ≤ 0.05.

For acute toxicity studies, data was collected using the Acentos preclinical software (PDS Inc.) and statistics were performed with the built-in function. Quantitative data is shown as mean ± standard deviation, unless otherwise mentioned. The data for males and females were separately analyzed for equal variance and normality. Data that were normally distributed and had equal variance were analyzed using Student’s *t* test. If the normality or variance assumptions were not met, the analysis was performed with the non-parametric Mann-Whitney rank sum test. Group differences were considered significant if *p* ≤ 0.05.

## Results

### Biodistribution and pharmacokinetics

**Fig. 3:**
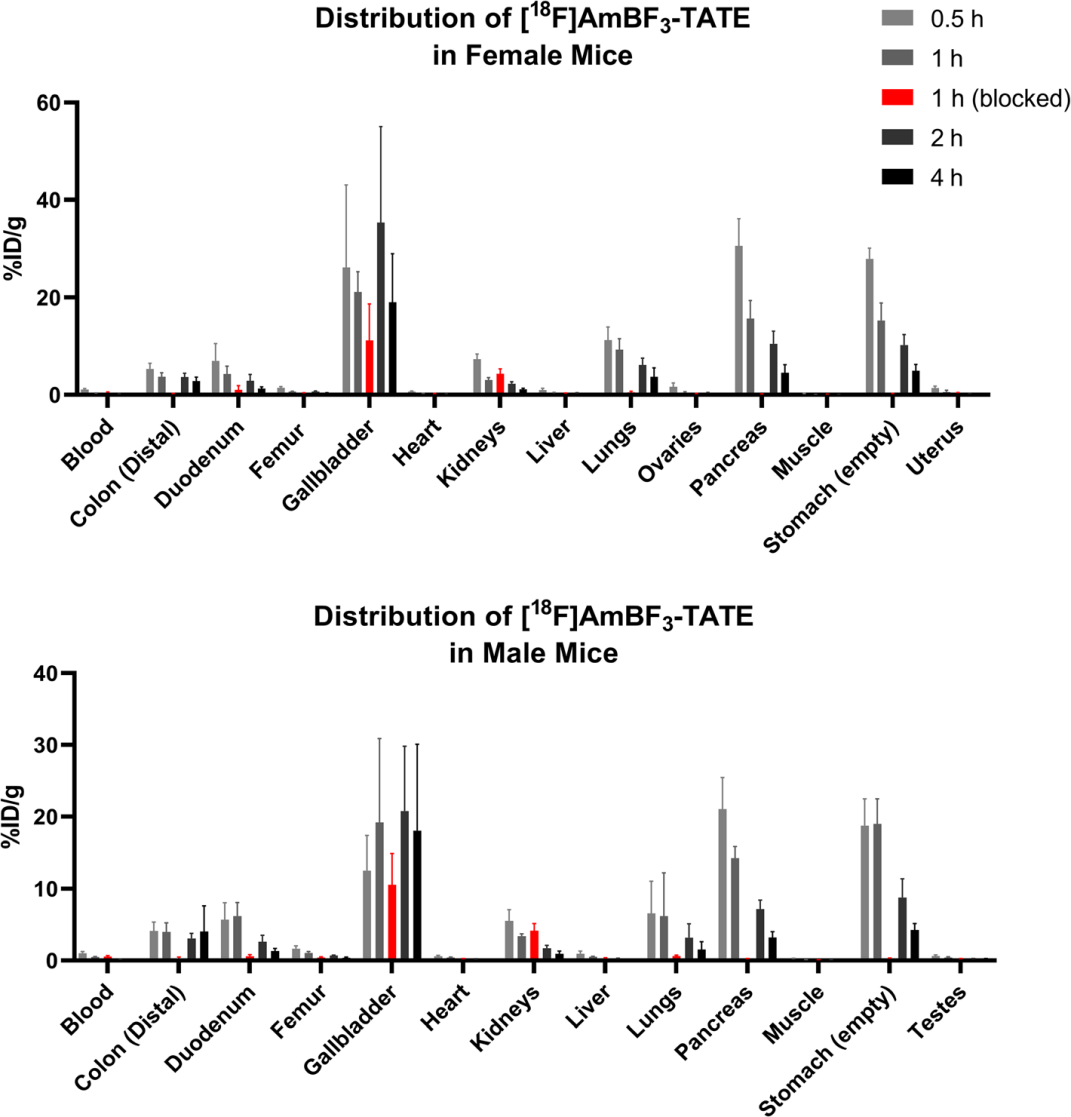
Distribution of [^18^F]AmBF_3_-TATE in mice at selected time points. Blocking was performed with pre-injection of 100 μg of [^19^F]AmBF_3_-TATE 30 min before radiopharmaceutical administration

The biodistribution data can be found in Fig. 3, Additional file 1: Tables S1 and S2. The organs with the highest specific uptake of [^18^F]AmBF_3_-TATE at the earliest examined time point (30 min post-injection (p.i.)) were the pancreas (F, 30.5 ± 5.64 %ID/g and M, 21.0 ± 4.42 %ID/g), stomach (F, 27.9 ± 2.20 %ID/g and M, 18.8 ± 3.73 %ID/g), and lungs (F, 11.2 ± 2.68 %ID/g and M, 6.56 ± 4.50 %ID/g). The radioactivity in these organs gradually decreased at subsequent time points. In non-target tissues, the uptake of [^18^F]AmBF_3_-TATE was negligible at most time points examined. [^18^F]AmBF_3_-TATE was rapidly cleared via the urinary and hepatobiliary tracts as evident by high and sustained levels of radioactivity in the urine at all time points examined (range, 105 ± 129 to 605 ± 716 %ID/g) and gall bladder (range, 12.5 ± 4.85 to 35.3 ± 19.8 %ID/g). The pharmacokinetics of [^18^F]AmBF_3_-TATE was very similar between the two sexes, with the noticeable exception of higher urinary bladder uptake in male mice at all time points.

To study target specificity of [^18^F]AmBF_3_-TATE, a group of animals were administered a blocking dose of [^19^F]AmBF_3_-TATE 30 min prior to radiopharmaceutical administration. For female mice, the uptake in the pancreas (15.7 ± 3.72 vs 0.13 ± 0.03 %ID/g; *p* < 0.001), stomach (15.3 ± 3.63 vs 0.23 ± 0.05 %ID/g; *p* < 0.001), and lungs (9.26 ± 2.24 vs 0.54 ± 0.16 %ID/g; *p* < 0.001) were significantly reduced at 1 h p.i. compared to non-blocked group. This was also observed for male mice, as the uptake in the pancreas (14.3 ± 1.61 vs 0.18 ± 0.09 %ID/g; *p* < 0.001), stomach (19.0 ± 3.49 vs 0.26 ± 0.08 %ID/g; *p* < 0.001), and lungs (6.17 ± 6.04 vs 0.61 ± 0.14 %ID/g; *p* = 0.02) were significantly reduced compared to non-blocked group.

### Dosimetry estimations

The absorbed doses in mice are shown in Additional file 1: Table S3, based on kinetic curves derived from biodistribution data (Additional file 1: Figs. S1 and S2). For both sexes, the organ that received the highest dose from [^18^F]AmBF_3_-TATE was the urinary bladder (F, 8.27 mGy/MBq and M, 10.5 mGy/MBq). This was followed by the pancreas (F, 2.05 mGy/MBq and M, 1.71 mGy/MBq), intestines (range, 1.36–1.45 mGy/MBq), and kidneys (F, 1.24 mGy/MBq and M, 1.13 mGy/MBq). Aside from target and excretory organs, all other organs received less than 1 mGy/MBq.

The estimated absorbed doses for an average adult human were extrapolated from mouse data and shown in Table 1. Consistent with the mouse model, the organ with the highest expected dose was the urinary bladder (F, 2.67 × 10^−2^ mGy/MBq and M, 3.02 × 10^−2^ mGy/MBq). This was followed by the pancreas (F, 2.80 × 10^−2^ mGy/MBq and M, 1.81 × 10^−2^ mGy/MBq), lungs (F, 1.27 × 10^−2^ mGy/MBq and M, 6.25 × 10^−3^ mGy/MBq), and stomach wall (F, 1.18 × 10^−2^ mGy/MBq and M, 8.14 × 10^−3^ mGy/MBq). The kidneys are expected to receive 6.69 × 10^−3^ mGy/MBq in females vs 4.78 × 10^−3^ mGy/MBq in males.

**Table 1:** Estimated absorbed doses [mSv/MBq] for different organs in the average adult calculated with OLINDA software

Target organ	[^18^F]AmBF_3_-TATE		[^68^Ga]Ga-DOTA-TATE*
	Female	Male	
Adrenals	5.87E−03	4.84E−03	1.46E−02
Brain	1.62E−04	1.61E−04	9.86E−03
Breast	7.73E−04	–	9.96E−03
Esophagus	2.10E−03	1.57E−03	–
Eye	2.43E−04	3.08E−04	–
Gallbladder wall	5.49E−03	3.68E−03	1.49E−02
Left colon	6.29E−03	6.71E−03	1.29E−02
Small intestine	8.43E−03	6.20E−03	1.38E−02
Stomach wall	1.18E−02	8.14E−03	1.38E−02
Right colon	4.29E−03	3.68E−03	1.29E−02
Rectum	5.91E−03	4.22E−03	–
Heart	2.81E−03	2.08E−03	1.23E−02
Kidneys	6.69E−03	4.78E−03	9.21E−02
Liver	2.33E−03	1.74E−03	4.50E−02
Lungs	1.27E−02	6.25E−03	1.15E−02
Ovaries	2.45E−03	–	1.31E−02
Pancreas	2.80E−02	1.81E−02	1.67E−02
Prostate	–	2.66E−03	–
Salivary glands	7.32E−04	9.24E−04	1.17E-02
Red marrow	1.28E−03	1.02E−03	9.61E−03
Skeleton	7.92E−04	7.13E−04	1.55E−02
Spleen	3.19E−03	2.07E−03	2.82E−01
Testes	–	1.11E−03	1.12E−02
Thymus	6.08E−03	4.20E−03	1.09E−02
Thyroid	1.99E−03	1.66E−03	1.87E−02
Urinary bladder	2.67E−02	3.02E−02	1.25E−01
Uterus	4.50E−03	–	1.47E−02^#^
Remainder of body	1.49E−03	1.04E−03	1.34E−02
Effective dose	5.91E−03	4.36E−03	2.57E−02

*Reproduced with permission from Walker et al. [13]

^#^Uterus dosimetry was estimated as all scanned subjects were male

### Acute toxicity

No mortalities occurred during the study. All interim study animals were euthanized on day 2, as scheduled, and all main study animals completed the 14-day treatment-free recovery period and were euthanized as scheduled on day 15 (injections of test and control agents performed on day 1). For clinical observations, one male in the main study exhibited alocepia on both forelimbs on day 14 of the study, while the rest of the animals appeared normal throughout. Body weight change and food consumption were measured and shown in Table 2. At the end of recovery period, males in the treated group exhibited significantly decreased body weight gain when compared to the control group (*p* < 0.05). There were no other statistically significant findings in body weight, body weight changes, or food consumption between the treated and control groups during the study.

**Table 2:** Summary of body weights, body weight changes, and food consumption during 14-day treatment-free recovery period

Group	Dose (mg/kg)	Mean body weight (g) ± SD				Body weight changes (g) ± SD Days 1–14 (*n* = 5)	Mean food consumption (g) ± SD Days 1–14 (*n* = 5)
		Day 1 (*n* = 15)	Day 2 (*n* = 10)	Day 8 (*n* = 5)	Day 14 (*n* = 5)		
1-F	0	212.3 ± 11.4	197.0 ± 10.9	232.4 ± 10.5	242.2 ± 13.2	+ 30.2 ± 8.9	230.6 ± 15.4
2-F	0.742	212.2 ± 13.6	195.4 ± 11.3	227.1 ± 23.6	240.9 ± 19.7	+ 29.3 ± 4.7	229.8 ± 16.9
1-M	0	295.5 ± 15.0	265.8 ± 14.1	359.7 ± 14.7	403.2 ± 21.1	+ 108.1 ± 14.8	404.0 ± 32.3
2-M	0.742	297.2 ± 17.2	265.4 ± 13.5	358.0 ± 21.1	388.5 ± 16.1	+ 86.6 ± 11.5*	365.7 ± 19.4

*Significantly different from control group (*p* < 0.05)

On occasion, individual values for hematology and clinical chemistry parameters were slightly outside the established normal reference ranges (data not shown). However, these values were also observed in the control group and therefore were considered incidental, not associated with other findings and of no toxicological significance. At the end of the interim study (day 2), treated males exhibited statistically significant decreases in several clinical chemistry parameters including serum albumin (*p* < 0.01), calcemia (*p* < 0.01), total protein (*p* < 0.05), and natremia (*p* < 0.01) when compared to the control group. The decreases were mild, ranging from 2.0% to 6.9%, and remained within the normal ranges except for natremia which was slightly above the normal range (136–143 mmol/L) in both the treated (144 mmol/L) and control groups (147 mmol/L). At the end of the main study (day 15), treated females exhibited decreased hematocrit (*p* < 0.01) and increased calcemia (*p* < 0.05) compared to the control group. These changes were mild, − 4.4% and + 2.9%, respectively, and remained within the normal ranges.

Aside from focal alopecia, gross pathology reported a treated rat had a cortical renal mass. Upon histopathological examination, it was interpreted that both findings were unrelated to treatment. Sporadic incidental findings of no general clinical consequence (inflammatory, developmental, degenerative, regenerative, proliferative) were observed histologically in small numbers of rats from both groups.

### Production of [18F]AmBF3-TATE on Trasis AllInOne Module

The three production runs had activity yields of 15.6 ± 4.59 GBq, decay corrected radiochemical yields of 15 ± 4%, molar activity of 435 ± 162 GBq/μmol, and radiochemical purity of 98.0 ± 1.73% (Table 3). The average synthesis time following target bombardment was 60 min. All batches of [^18^F]AmBF_3_-TATE passed the QC parameters listed in Table 3. In terms of residual solvents, the average ppm value for ethanol was 44400 ± 7100. Residual acetonitrile (< 10 ppm), dimethylformamide (< 1 ppm), and pyridazine (< 1 ppm) were undetected or below the limit of quantification (LOQ). The concentration of ascorbic acid present in the formulation was 1000 mg/L. The formulations were sterile as no microbial contamination was observed in delayed testing. All additional tests were qualitatively passed or below LOQs. The representative radio-HPLC chromatograms of [^18^F]AmBF_3_-TATE are shown in Additional file 1: Figs. S3 and S4. [^18^F]AmBF_3_-TATE was stable for up to 6 h after end of synthesis (EOS) (Additional file 1: Fig. S5).

**Table 3:** Quality control (QC) of [^18^F]AmBF_3_-TATE injection solution from three individual validation runs

Run ID			V-ABT20190417A	V-ABT20190430A	V-ABT20190508A
Batch size (GBq)			12.669 GBq	13.240 GBq	20.890 GBq
Parameters	Method	Acceptance criteria	Results		
Filter integrity	Bubble point	> 50 psi	Pass	Pass	Pass
Appearance	Visual	Clear, colorless to slightly yellowish solution; free of visible particulates	Pass	Pass	Pass
pH	pH indicator strip	4.5–7.5	5.5	5.5	5.5
Residual solvents	GC-FID	Ethanol: < 100,000 ppm	36,743 ppm	50,820 ppm	45,641 ppm
		Acetonitrile: < 400 ppm	< 10 ppm	< 10 ppm	< 10 ppm
	HPLC-UV-radiometric	Dimethylformamide (DMF): < 880 ppm	< 1 ppm	< 1 ppm	< 1 ppm
		Pyridazine: < 400 ppm	< 1 ppm	< 1 ppm	< 1 ppm
	Dose calibrator	Half-life: 105–115 min	112 min	109 min	110 min
Radiochemical identification	HPLC-UV-Radiometric	*t*_R_ of [^18^F]AMBF_3_-TATE: 100 ± 10% of the reference standard	100%	100%	100%
Radiochemical purity	HPLC-UV-radiometric	≥ 90%	99%	99%	96%
Molar activity	HPLC-UV-radiometric	≥ 44 GBq/μmol @ EOS	496 GBq/μmol	251 GBq/μmol	559 GBq/μmol
Ascorbic acid	Colorimetric assay	< 20,000 mg/L	1000 mg/L	1000 mg/L	1000 mg/L
Bacterial endotoxins	LAL test	< 10.0 EU/mL	< 10.0 EU/mL	< 10.0 EU/mL	< 10.0 EU/mL
Radionuclidic purity^1^	Gamma spectroscopy	≥ 99.5%	100%	100%	100%
Sterility^1^	Post-release	Sterile, no growth	Pass	Pass	Pass

*GC-FID* gas chromatography flame ionization detector, *t*_*R*_ retention time, *EOS* end of synthesis, *LAL* limulus amebocyte lysate

^1^These tests are done retrospectively

## Discussion

The success of radiolabelled somatostatin analogs has been at the forefront of personalized medicine in nuclear medicine [14]. By changing the radioisotope of choice, SSTR2 ligands can be used effectively to diagnose, treat, and monitor response to treatment for NET patients. In the present study, we reported the biodistribution, pharmacokinetics, dosimetry, and automated production of [^18^F]AmBF_3_-TATE in anticipation for clinical translation.

We conducted a GLP-biodistribution study in ICR mice. Consistent with our previous studies, [^18^F]AmBF_3_-TATE was cleared through the urinary and hepatobiliary tracts as evidenced by high and sustained levels of radioactivity in urine and gallbladder [8]. In male mice, higher urinary bladder uptake was observed at all time points. Organs that express SSTR2 including pancreas, lungs, and stomach showed high specific uptake of [^18^F]AmBF_3_-TATE as early as 30 min p.i. Pre-blocking with [^19^F]AmBF_3_-TATE significantly reduced uptake in SSTR2-expressing organs to near background levels, demonstrating target specificity of [^18^F]AmBF_3_-TATE. Unlike the previous study, we measured radiotracer uptake in stomach by emptying contents prior to counting. The uptake of [^18^F]AmBF_3_-TATE in non-target tissues was negligible at most time points. Since free ^18^F-fluoride accumulates in the bone, uptake in the femur was used as a surrogate to determine defluorination *in vivo*. The uptake in the femur was 1.45–1.66 %ID/g at 30 min p.i. and decreased continuously to 0.34–0.38 %ID/g at 4 h p.i., indicating the product was stable against defluorination.

Based on dosimetry estimations, the organs that are expected to receive the highest absorbed doses are excretory organs (urinary bladder and intestines) or those that express SSTR2 (pancreas, lungs, and stomach). The estimated effective doses for [^18^F]AmBF_3_-TATE for the average adult female and male are 5.91E−03 and 4.36E−03 mSv/MBq, respectively. Based on preclinical PET images of three mice, Lisova et al. calculated that the effective dose for [^18^F]AmBF_3_-TATE to be 1.16E-02 to 1.26E-2 mSv/MBq [15], which is approximately 2–3 folds higher. Given the differences in study design and sample size (*n* = 80 in present study), we believe our current study offers an accurate representation of [^18^F]AmBF_3_-TATE pharmacokinetics especially in organs that are difficult to identify/demarcate in preclinical PET images. For example, the dosimetry estimation for lungs in Lisova et al. may be underestimated due to cell/tissue density, partial volume effect and motion artifacts. As shown in Additional file 1: Tables S1 and S2, this organ expresses SSTR2 and uptake can clearly be blocked with pre-injection of the nonradioactive standard. From literature, the estimated effective doses for [^68^Ga]Ga-DOTA-TATE and 2-deoxy-2-[^18^F]fluoro-d-glucose (FDG) are 2.57 E−02 and 1.92E−02 mSv/MBq, respectively [13]. A patient receiving a 370 MBq dose of [^18^F]AmBF_3_-TATE would receive between 1.61 and 2.18 mSv. In comparison, patients will receive 4.75 mSv from [^68^Ga]Ga-DOTA-TATE (185 MBq) or 7.02 mSv from FDG (370 MBq). It is important to note that the effective dose for [^18^F]AmBF_3_-TATE was extrapolated from mouse data, while the values provided for [^68^Ga]Ga-DOTA-TATE and FDG are derived from patient scans.

Acute toxicity of [^19^F]AmBF_3_-TATE was assessed in Sprague-Dawley rats in adherence to ICH guidelines. Rats were intravenously administered a single dose of [^19^F]AmBF_3_-TATE at 0.742 mg/kg and monitored for up to 14 days. The evaluated dose was 1000× the anticipated clinical dose, based on setting molar activity limit as ≥ 9.25 GBq/μmol with a 370 MBq dose for a 70-kg patient at time of injection. No test-item–related effects were observed during the GLP toxicity study, upon evaluation of clinical observations, body weights, food consumption, clinical pathology, gross pathology, and histopathology. Weight gain was observed for all mice over the 14-day recovery period; however, males in the treated group exhibited significantly lower body weight gain compared to the control group. Peripheral administration of somatostatin can reduce food intake in rats [16, 17] and inhibits pituitary hormone secretion, including growth and adrenocorticotropic hormone secretion [18]; however, it is unclear why only males were affected in the present study. All other findings from clinical observations, hematology, clinical chemistry, gross pathology, and histopathology were considered incidental and of no clinical consequence. The data suggests [^19^F]AmBF_3_-TATE to be safe for administration.

We automated the radiolabeling process on a Trasis AllInOne module for good manufacturing practice (GMP)-compliant production. [^18^F]AmBF_3_-TATE was produced in good activity yields (12.7 to 20.9 GBq) and high average molar activity (251 to 559 GBq/μmol), with each synthesis run taking 60 min on average. Based on a calculation of 370 MBq/dose and accounting for decay and 30 min in between patient injections with a single scanner setup, we estimate this would amount to 8–10 individual patient doses from one production run. For the automation process, no additional effort was spent on reducing purification time or on optimizing radiochemical yield. We expect the activity yields will satisfy clinical demands; however, it is possible to further improve yield and/or molar activity by starting with higher levels of ^18^F-fluoride radioactivity from our experience with manual synthesis. All three validation runs of [^18^F]AmBF_3_-TATE passed QC parameters. Ascorbic acid was added to the formulation in order to prevent radiolysis of the radiopharmaceutical. Accordingly, no degradation of [^18^F]AmBF_3_-TATE in formulation was observed for up to 6 h.

The impact of [^18^F]AmBF_3_-TATE would be significant; the longer half-life of ^18^F will enable distribution of the compound to centers that have no access to a cyclotron or a ^68^Ge/^68^Ga generator. For facilities that have a cyclotron, ^18^F would eliminate the need for purchasing an expensive generator that has a limited lifespan and elution capacity per day. Moreover, multiple patient doses can be prepared for [^18^F]AmBF_3_-TATE from a single production run, instead of two to four doses per batch for ^68^Ga-based somatostatin analogs. For daily clinical logistics, the superior half-life also allows greater freedom in the scheduling of patients and for dealing with unforeseen events that can delay imaging. Another important impact of the radiopharmaceutical lies in its superior imaging characteristics that could improve PET imaging accuracy of NETs. Due to a shorter positron range which allows for higher spatial resolution, improved receptor affinity, and low liver retention, we anticipate that [^18^F]AmBF_3_-TATE will be equal or superior to current ^68^Ga-labeled agents to detect small metastases. Moreover, the automated setup for production presented can be reused in centers with similar equipment, further facilitating access to the radiopharmaceutical, as no training in manual synthesis would be necessary.

The implementation of an ^18^F-labeled SSTR2 imaging radioligand can improve access to quality imaging for NET patients. A clinical trial application is planned to assess the safety and radiation dosimetry of [^18^F]AmBF_3_-TATE in NET patients.

## Conclusions

[^18^F]AmBF_3_-TATE is a promising SSTR2 targeting pharmaceutical. In GLP studies with small animals, [^18^F]AmBF_3_-TATE showed rapid clearance from non-target tissues leading to favorable dosimetry. [^19^F]AmBF_3_-TATE was well tolerated at 1000× clinical dose, suggesting an excellent safety profile. Lastly, the synthesis of [^18^F]AmBF_3_-TATE was automated to comply with regulatory guidelines for clinical translation.

## Supplementary information

**Additional file 1: Table S1.** Biodistribution of [^18^F]AmBF_3_-TATE in ICR female mice at selected time points (*n* = 8 per group). Values reported in %ID/g. **Table S2.** Biodistribution of [^18^F]AmBF_3_-TATE in ICR male mice at selected time points (*n* = 8 per group). Values reported in %ID/g. **Table S3.** OLINDA-calculated dosimetry [mSv/MBq] using the 25g MOBY mouse phantom from biodistribution data. **Figure S1.** Uptake of [^18^F]AmBF_3_-TATE in female mice as a function of time for the bladder, blood, kidneys, liver, and pancreas. **Figure S2.** Uptake of [^18^F]AmBF_3_-TATE in male mice as a function of time for the bladder, blood, kidneys, liver, and pancreas. **Figure S3.** Radio-chromatogram of [^18^F]AmBF_3_-TATE acquired by the integrated HPLC system on the Trasis AllInOne module. **Figure S4.** QC Radio-chromatogram of [^18^F]AmBF_3_-TATE acquired by the Agilent HPLC system. **Figure S5.** Radio-chromatogram of [^18^F]AmBF_3_-TATE acquired by the Agilent HPLC system 6 h after EOS.

## Data Availability

The datasets used and/or analysed during the current study are available from the corresponding author on reasonable request.

## Abbreviations

DMF: Dimethylformamide EOS End of synthesis FDA US Food and Drug Administration GLP Good laboratory practice GMP Good manufacturing practice H&E Hematoxylin and eosin ID/g Injected dose per gram of tissue LOQ Limit of quantification NET Neuroendocrine tumor PBS Phosphate-buffered saline PET Positron emission tomography p.i. Post-injection QC Quality control SNMMI Society of Nuclear Medicine and Molecular Imaging SSTR2 Somatostatin receptor subtype 2 TATE Octreotate
