# Supplementary material for: Pharmacokinetics, radiation dosimetry, acute toxicity and automated synthesis of [18F]AmBF3-TATE
Source: EJNMMI Res. 2020 Mar 19;10:25. doi: 10.1186/s13550-020-0611-9 (PMC7080905; doi:10.1186/s13550-020-0611-9)
Supplement: Supplementary file 1 — Additional file 1: Table S1. Biodistribution of [18F]AmBF3-TATE in ICR female mice at selected time points (n = 8 per group). Values reported in %ID/g. Table S2. Biodistribution of [18F]AmBF3-TATE in ICR male mice at selected time points (n = 8 per group). Values reported in %ID/g. Table S3. OLINDA-calculated dosimetry [mSv/MBq] using the 25g MOBY mouse phantom from biodistribution data. Figure S1. Uptake of [18F]AmBF3-TATE in female mice as a function of time for the bladder, blood, kidneys, liver, and pancreas. Figure S2. Uptake of [18F]AmBF3-TATE in male mice as a function of time for the bladder, blood, kidneys, liver, and pancreas. Figure S3. Radio-chromatogram of [18F]AmBF3-TATE acquired by the integrated HPLC system on the Trasis AllInOne module. Figure S4. QC Radio-chromatogram of [18F]AmBF3-TATE acquired by the Agilent HPLC system. Figure S5. Radio-chromatogram of [18F]AmBF3-TATE acquired by the Agilent HPLC system 6 h after EOS. [file 13550_2020_611_MOESM1_ESM.docx]

**SUPPLEMENTAL INFORMATION**

**Pharmacokinetics, radiation dosimetry, acute toxicity and automated synthesis of [^18^F]AmBF_3_-TATE**

Joseph Lau^1^, Jinhe Pan^1,2^, Etienne Rousseau^1^, Carlos F. Uribe^1^, Sudhakara Reddy Seelam^2^, Brent W. Sutherland^3^, David M. Perrin^4^, Kuo-Shyan Lin^1,2^, François Bénard^1,2^

^1^Department of Molecular Oncology, BC Cancer, Vancouver BC, Canada

^2^Department of Functional Imaging, BC Cancer, Vancouver BC, Canada

^3^Department of Experimental Therapeutics, BC Cancer, Vancouver BC, Canada

^4^Department of Chemistry, University of British Columbia, Vancouver BC, Canada

**Contents**

1. Supplemental Tables 1-3
2. Supplemental Figures 1-5

**Supplemental Table 1.** Biodistribution of [^18^F]AmBF_3_-TATE in ICR female mice at selected time points (n=8 per group). Values reported in %ID/g.

|  | **30 min** | | **1 h** | | **1 h blocked*** | | **2 h** | | **4 h** | |
| --- | --- | --- | --- | --- | --- | --- | --- | --- | --- | --- |
|  | **Mean** | **SD** | **Mean** | **SD** | **Mean** | **SD** | **Mean** | **SD** | **Mean** | **SD** |
| Adrenal Gland (2×) | 4.06 | 1.2 | 1.67 | 0.84 | 0.15 | 0.06 | 2.36 | 0.69 | 1.18 | 0.23 |
| Bladder | 3.7 | 1.66 | 2.91 | 1.63 | 3.18 | 2.31 | 2.27 | 3.22 | 1.43 | 2.09 |
| Blood | 1.08 | 0.2 | 0.34 | 0.05 | 0.45 | 0.12 | 0.24 | 0.06 | 0.15 | 0.13 |
| Brain | 0.07 | 0.06 | 0.02 | 0 | 0.02 | 0.01 | 0.02 | 0 | 0.01 | 0.01 |
| Cecum (empty) | 2.94 | 0.23 | 1.96 | 0.27 | 0.17 | 0.03 | 2.14 | 0.55 | 4.01 | 0.93 |
| Colon (distal) | 5.33 | 1.16 | 3.67 | 0.85 | 0.21 | 0.09 | 3.64 | 0.78 | 2.81 | 0.81 |
| Colon (proximal) | 5.64 | 2.16 | 3.23 | 1.3 | 0.19 | 0.07 | 2.81 | 0.99 | 4.42 | 2.57 |
| Duodenum | 6.99 | 3.52 | 4.3 | 1.58 | 1.02 | 0.88 | 2.88 | 1.28 | 1.25 | 0.38 |
| Femur (1×) | 1.45 | 0.22 | 0.66 | 0.08 | 0.34 | 0.07 | 0.66 | 0.12 | 0.34 | 0.09 |
| Gallbladder | 26.05 | 17.01 | 21.13 | 4.11 | 11.16 | 7.53 | 35.32 | 19.75 | 19 | 9.92 |
| Heart | 0.66 | 0.11 | 0.3 | 0.04 | 0.18 | 0.07 | 0.24 | 0.04 | 0.14 | 0.02 |
| Kidney (both) | 7.33 | 1.05 | 3 | 0.54 | 4.31 | 1.03 | 2.28 | 0.41 | 1.1 | 0.23 |
| Liver | 0.97 | 0.36 | 0.4 | 0.05 | 0.29 | 0.07 | 0.34 | 0.1 | 0.17 | 0.04 |
| Lungs | 11.23 | 2.68 | 9.26 | 2.24 | 0.54 | 0.16 | 6.13 | 1.41 | 3.65 | 1.86 |
| Ovary (both) | 1.65 | 0.77 | 0.49 | 0.12 | 0.19 | 0.07 | 0.36 | 0.12 | 0.14 | 0.05 |
| Pancreas | 30.5 | 5.64 | 15.67 | 3.72 | 0.13 | 0.03 | 10.48 | 2.61 | 4.51 | 1.66 |
| Salivary Gland (2×) | 0.81 | 0.25 | 0.32 | 0.11 | 0.15 | 0.04 | 0.21 | 0.08 | 0.12 | 0.04 |
| Skeletal Muscle (L. femoral) | 0.23 | 0.04 | 0.06 | 0.01 | 0.08 | 0.03 | 0.05 | 0.01 | 0.02 | 0.01 |
| Skin (1cm^2^) | 0.95 | 0.4 | 0.25 | 0.07 | 0.28 | 0.09 | 0.17 | 0.11 | 0.08 | 0.03 |
| Spleen | 1.22 | 0.45 | 0.55 | 0.17 | 0.18 | 0.04 | 0.45 | 0.23 | 0.28 | 0.1 |
| Stomach (empty) | 27.89 | 2.2 | 15.27 | 3.63 | 0.23 | 0.05 | 10.23 | 2.12 | 4.91 | 1.3 |
| Tail (3 pieces) | 3.67 | 1.52 | 1.51 | 0.33 | 1.01 | 0.52 | 0.71 | 0.16 | 0.47 | 0.2 |
| Thymus | 6.57 | 1.29 | 3.17 | 0.4 | 0.14 | 0.05 | 2.13 | 0.53 | 0.68 | 0.24 |
| Thyroid gland | 1.44 | 0.66 | 0.73 | 0.1 | 0.27 | 0.05 | 0.72 | 0.25 | 0.29 | 0.06 |
| Trachea | 1.54 | 0.86 | 0.78 | 0.38 | 0.34 | 0.16 | 1.69 | 1.22 | 0.29 | 0.26 |
| Urine | 605.45 | 715.58 | 151.32 | 106.45 | 253.24 | 259.3 | 245.25 | 250.88 | 105.27 | 129.16 |
| Uterus | 1.39 | 0.37 | 0.55 | 0.39 | 0.35 | 0.11 | 0.16 | 0.19 | 0.19 | 0.06 |

*Blocking group received 100 µg of [^19^F]AmBF_3_-TATE 30 min before radiopharmaceutical administration.

**Supplemental Table 2.** Biodistribution of [^18^F]AmBF_3_-TATE in ICR male mice at selected time points (n=8 per group). Values reported in %ID/g.

|  | **30 min** | | **1 h** | | **1 h blocked*** | | **2 h** | | **4 h** | |
| --- | --- | --- | --- | --- | --- | --- | --- | --- | --- | --- |
|  | **Mean** | **SD** | **Mean** | **SD** | **Mean** | **SD** | **Mean** | **SD** | **Mean** | **SD** |
| Adrenal Gland (2×) | 3.26 | 1.36 | 3.33 | 1.29 | 0.51 | 0.64 | 2.05 | 0.89 | 1.5 | 0.51 |
| Bladder | 86.24 | 43.72 | 77.13 | 47.41 | 81.21 | 67.02 | 72.62 | 70.58 | 43.09 | 42.23 |
| Blood | 1.01 | 0.25 | 0.47 | 0.07 | 0.55 | 0.13 | 0.18 | 0.03 | 0.09 | 0.01 |
| Brain | 0.05 | 0.02 | 0.03 | 0.01 | 0.03 | 0.01 | 0.02 | 0.02 | 0.02 | 0.01 |
| Cecum (empty) | 2.22 | 0.97 | 2.52 | 0.45 | 0.26 | 0.14 | 2.3 | 0.7 | 4.84 | 1.28 |
| Colon (Distal) | 4.12 | 1.24 | 3.97 | 1.27 | 0.31 | 0.16 | 3.07 | 0.71 | 4.05 | 3.58 |
| Colon (Proximal) | 5.26 | 1.51 | 4.96 | 1.6 | 0.37 | 0.41 | 3.64 | 0.73 | 5.87 | 3.32 |
| Duodenum | 5.67 | 2.38 | 6.19 | 1.89 | 0.57 | 0.25 | 2.61 | 0.92 | 1.33 | 0.34 |
| Femur (1×) | 1.66 | 0.4 | 1.04 | 0.21 | 0.42 | 0.09 | 0.68 | 0.08 | 0.38 | 0.1 |
| Gallbladder | 12.53 | 4.85 | 19.18 | 11.69 | 10.54 | 4.37 | 20.79 | 9.04 | 18.04 | 12.04 |
| Heart | 0.57 | 0.13 | 0.41 | 0.06 | 0.21 | 0.07 | 0.23 | 0.03 | 0.16 | 0.02 |
| Kidney (both) | 5.5 | 1.57 | 3.39 | 0.32 | 4.14 | 1.02 | 1.7 | 0.41 | 0.94 | 0.38 |
| Liver | 0.94 | 0.38 | 0.51 | 0.08 | 0.32 | 0.06 | 0.28 | 0.05 | 0.14 | 0.02 |
| Lungs | 6.56 | 4.5 | 6.17 | 6.04 | 0.61 | 0.14 | 3.18 | 1.94 | 1.51 | 1.09 |
| Pancreas | 21.04 | 4.42 | 14.26 | 1.61 | 0.18 | 0.09 | 7.14 | 1.29 | 3.21 | 0.81 |
| Salivary Gland (2×) | 0.93 | 0.21 | 0.64 | 0.2 | 0.2 | 0.04 | 0.39 | 0.08 | 0.26 | 0.04 |
| Skeletal Muscle (L. femoral) | 0.23 | 0.08 | 0.09 | 0.02 | 0.11 | 0.02 | 0.06 | 0.06 | 0.03 | 0.02 |
| Skin (1cm^2^) | 0.86 | 0.26 | 0.32 | 0.08 | 0.4 | 0.12 | 0.25 | 0.29 | 0.07 | 0.01 |
| Spleen | 0.99 | 0.38 | 1.05 | 0.35 | 0.25 | 0.06 | 0.45 | 0.09 | 0.29 | 0.1 |
| Stomach (empty) | 18.76 | 3.73 | 18.99 | 3.49 | 0.26 | 0.08 | 8.78 | 2.59 | 4.26 | 0.89 |
| Tail (3 pieces) | 2.31 | 0.47 | 1.56 | 0.74 | 0.97 | 0.17 | 0.65 | 0.13 | 0.38 | 0.07 |
| Testis (both) | 0.6 | 0.17 | 0.43 | 0.09 | 0.22 | 0.07 | 0.21 | 0.07 | 0.17 | 0.12 |
| Thymus | 4.67 | 0.95 | 3.53 | 0.42 | 0.21 | 0.11 | 1.74 | 0.46 | 0.64 | 0.14 |
| Thyroid gland | 1.28 | 0.23 | 0.93 | 0.19 | 0.56 | 0.35 | 0.51 | 0.12 | 0.37 | 0.08 |
| Trachea | 1.43 | 0.48 | 1.07 | 0.38 | 0.65 | 0.28 | 0.73 | 0.43 | 0.46 | 0.37 |
| Urine | 498.89 | 315.45 | 333.41 | 110.85 | 197.83 | 114.67 | 170.27 | 82.84 | 151.01 | 90.73 |

*Blocking group received 100 µg of [^19^F]AmBF_3_-TATE 30 min before radiopharmaceutical administration.

**Supplemental Table 3.** OLINDA-calculated dosimetry [mSv/MBq] using the 25g MOBY mouse phantom from biodistribution data.

| **Target organ** | **Female** | **Male** |
| --- | --- | --- |
| Brain | 1.28E-01 | 1.42E-01 |
| Large intestine | 1.41E+00 | 1.45E+00 |
| Small intestine | 1.38E+00 | 1.36E+00 |
| Stomach wall | 1.12E+00 | 9.73E-01 |
| Heart | 4.18E-01 | 3.92E-01 |
| Kidneys | 1.24E+00 | 1.13E+00 |
| Liver | 7.04E-01 | 6.54E-01 |
| Lungs | 4.69E-01 | 4.09E-01 |
| Pancreas | 2.05E+00 | 1.71E+00 |
| Skeleton | 6.52E-01 | 7.34E-01 |
| Spleen | 9.48E-01 | 8.31E-01 |
| Testes* | 8.14E-01 | 1.01E+00 |
| Thyroid | 2.45E-01 | 2.66E-01 |
| Urinary bladder | 8.27E+00 | 1.05E+01 |
| Rest of the body | 6.00E-01 | 6.21E-01 |

*Note: The MOBY phantom does not differentiate between sexes.

**
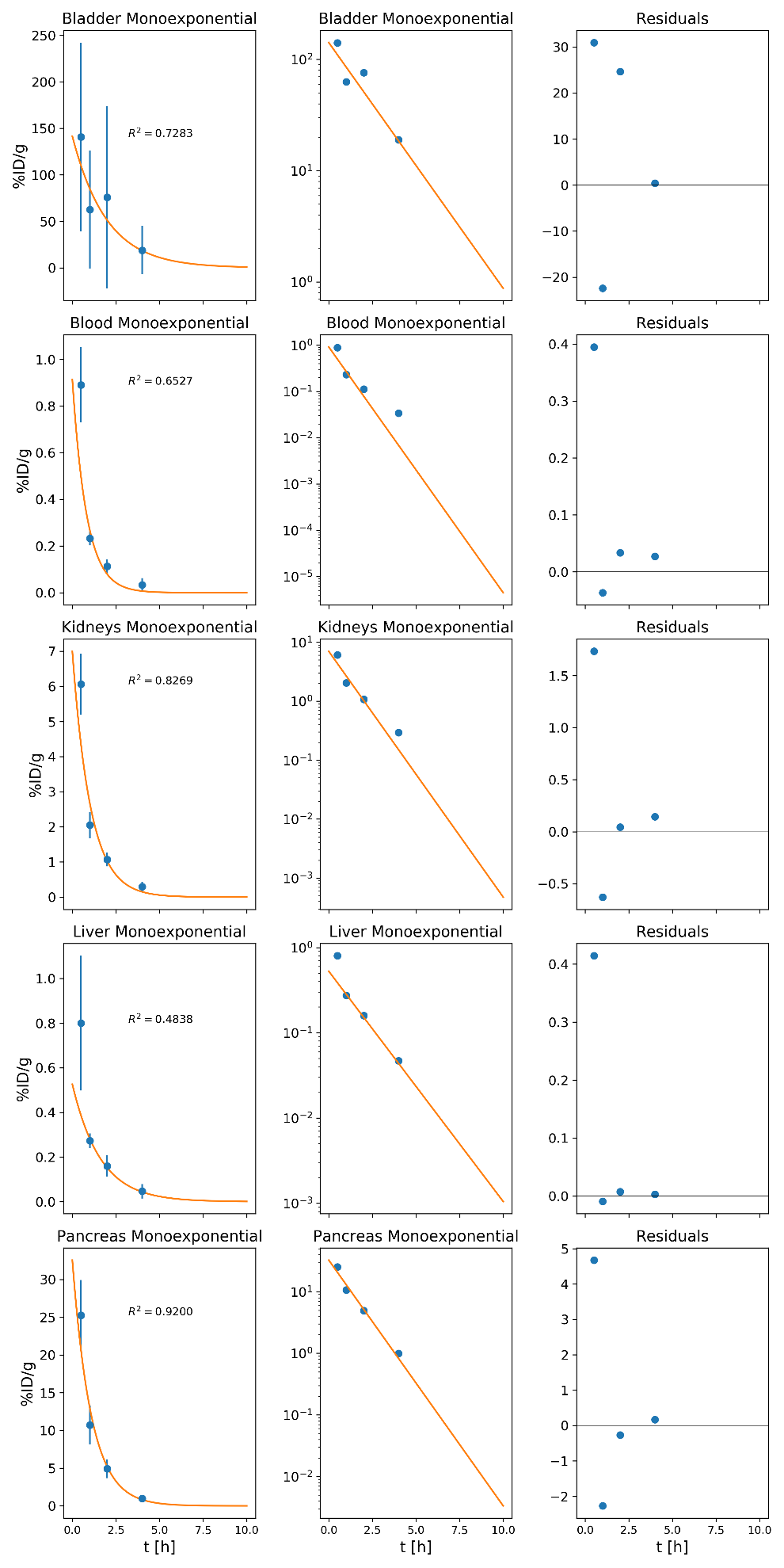
**

**Supplemental Figure 1.** Uptake of [^18^F]AmBF_3_-TATE in female mice as a function of time for bladder, blood, kidneys, liver and pancreas. The total number of decays per unit injected dose is calculated by multiplying the area under the curve by the phantom organ mass.

**
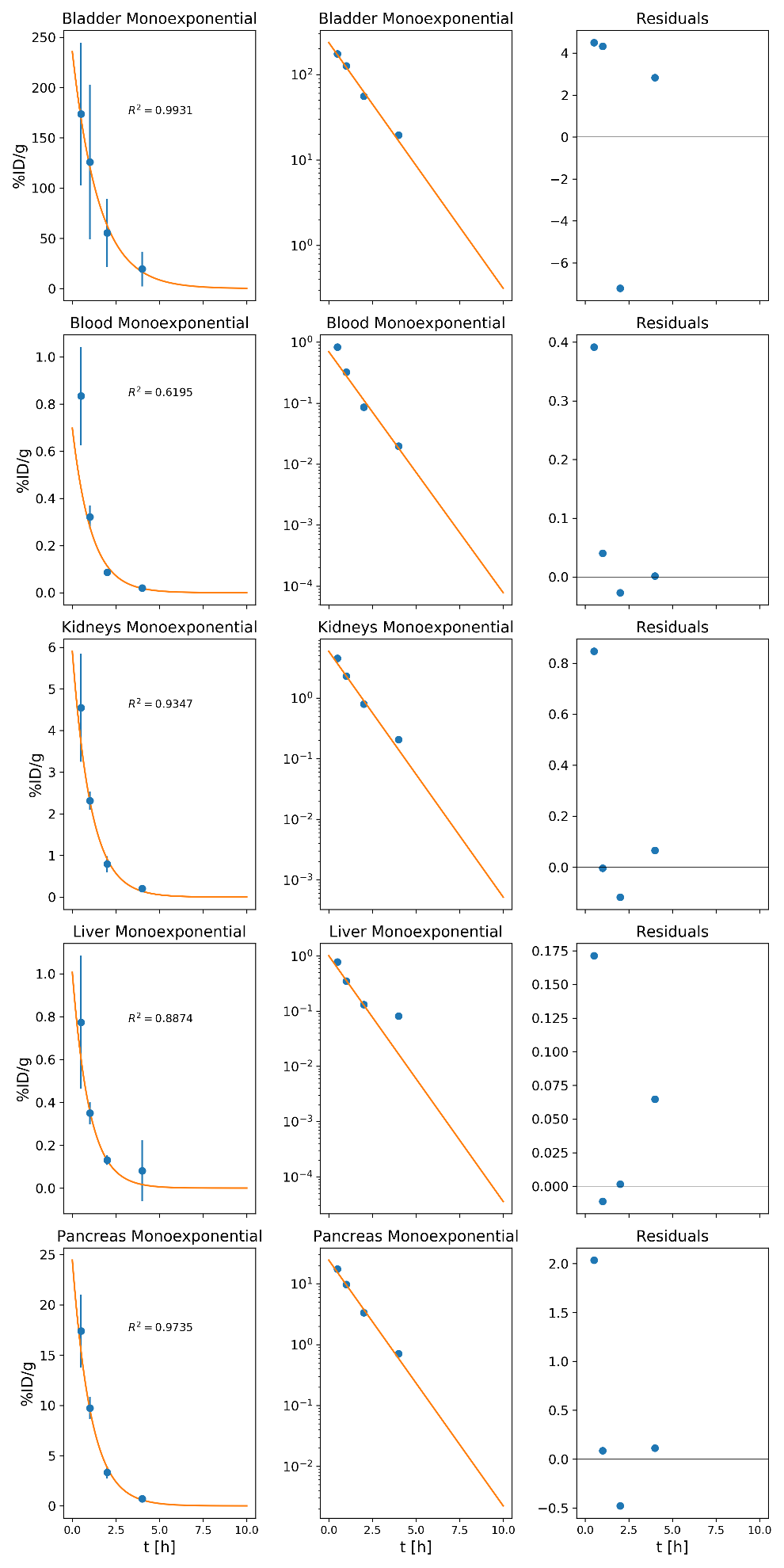
**

**Supplemental Figure 2.** Uptake of [^18^F]AmBF_3_-TATE in male mice as a function of time for bladder, blood, kidneys, liver and pancreas. The total number of decays per unit injected dose is calculated by multiplying the area under the curve by the phantom organ mass.


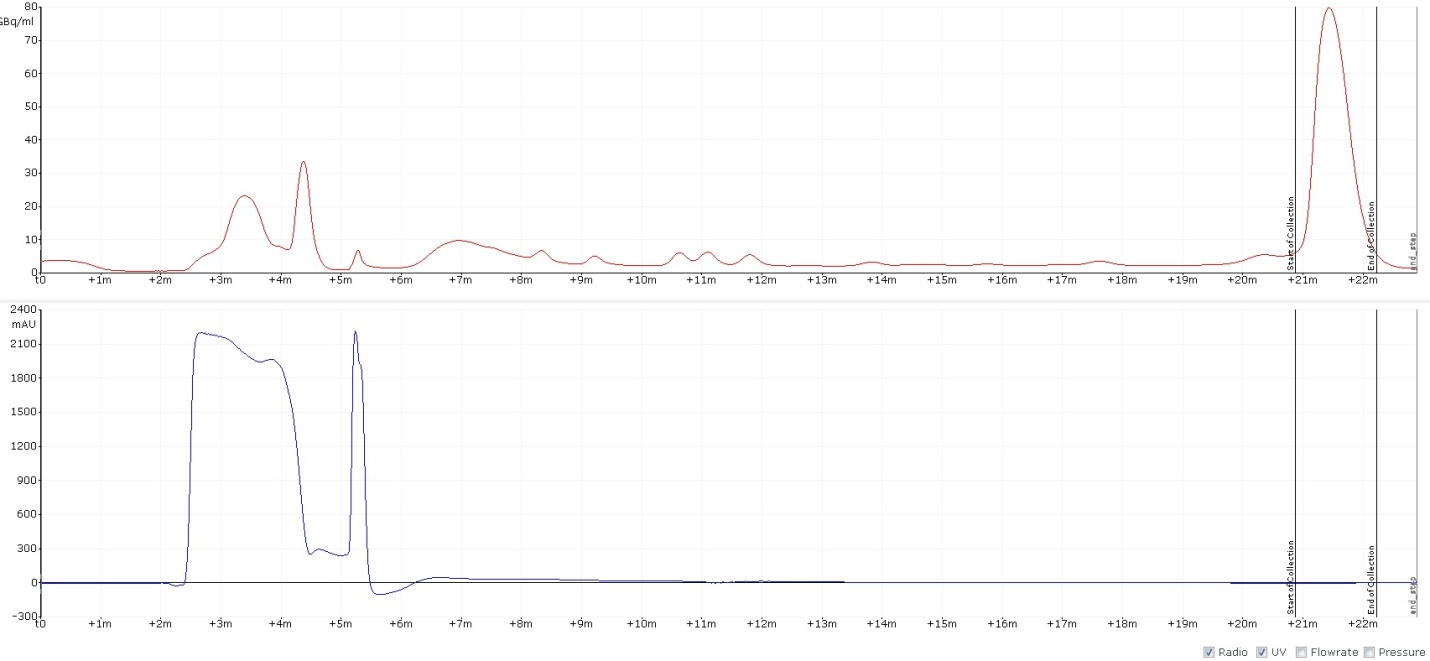


**Supplemental Figure 3**. Radio-chromatogram of [^18^F]AmBF_3_-TATE acquired by the integrated HPLC system on the Trasis AllInOne module. Top panel: radioactivity; bottom panel: UV absorbance at 254 nm.


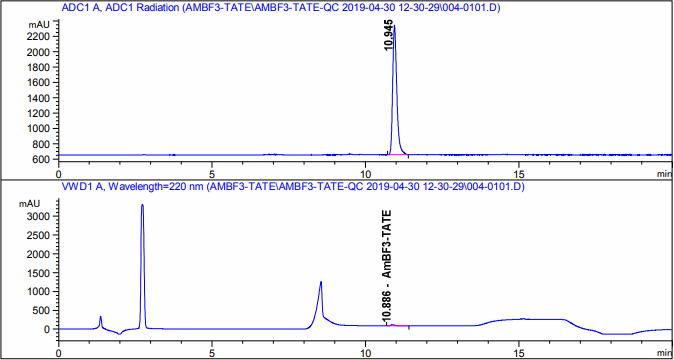


**Supplemental Figure** **4**. QC Radio-chromatogram of [^18^F]AmBF_3_-TATE acquired by the Agilent HPLC system. Top panel: radioactivity; bottom panel: UV absorbance at 220 nm. The peak at 8.5 min is part of the solvent front corresponding to a gradient change.


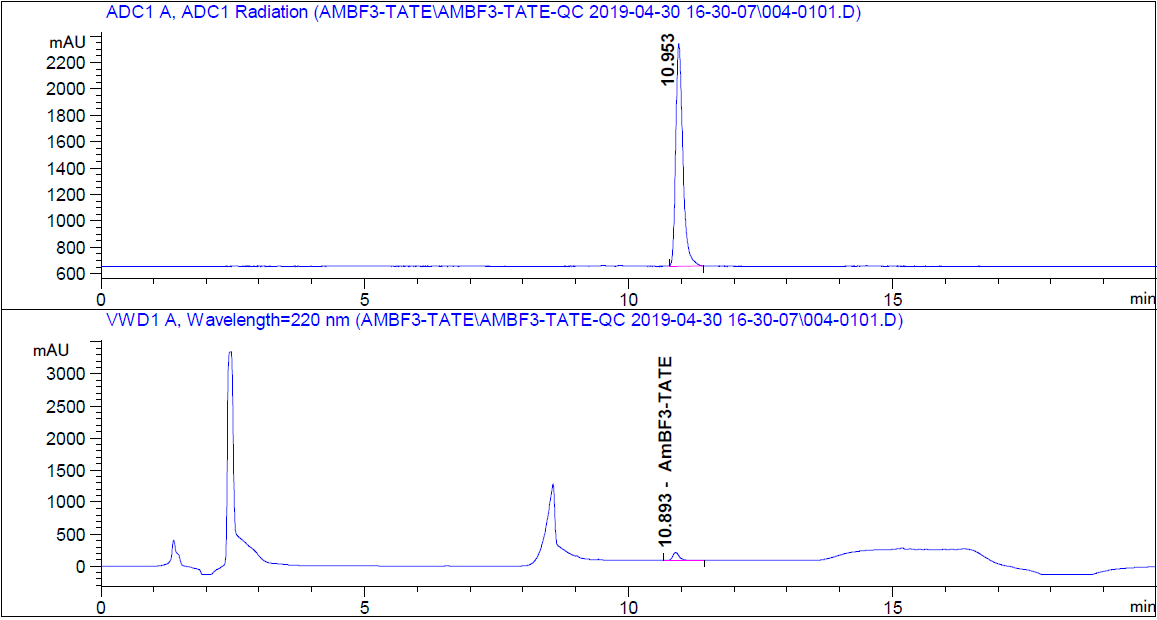


**Supplemental Figure** **5**. Radio-chromatogram of [^18^F]AmBF_3_-TATE acquired by the Agilent HPLC system 6 hours after EOS. Top panel: radioactivity; bottom panel: UV absorbance at 220 nm. No degradation of [^18^F]AmBF_3_-TATE was observed.
